# Supplementary material for: Strain distribution in WS2 monolayers detected through polarization-resolved second harmonic generation
Source: Sci Rep. 2024 Jul 2;14:15159. doi: 10.1038/s41598-024-66065-2 (PMC11219737; doi:10.1038/s41598-024-66065-2)
Supplement: Supplementary file 1 — Supplementary Information. [file 41598_2024_66065_MOESM1_ESM.pdf]

Supplementary Information

Strain distribution in WS_2 monolayers detected through Polarization-resolved Second Harmonic Generation

George Kourmoulakis[1,2], Sotiris Psilodimitrakopoulos[1]*, George Miltos Maragkakis[1,3], Leonidas Mouchliadis[1], Antonios Michail[4,5], Joseph A Christodoulides[6], Manoj Tripathi[7], Alan B Dalton[7], John Parthenios[5], Konstantinos Papagelis[5,8], Emmanuel Stratakis[1,3]*, and George Kioseoglou[1,2]*

[1] Institute of Electronic Structure and Laser, Foundation for Research and Technology - Hellas, Heraklion, 71110, Crete, Greece
[2] Department of Materials Science and Technology, University of Crete, Heraklion, 70013 Crete, Greece
[3] Department of Physics, University of Crete, Heraklion Crete 70013, Greece
[4] Department of Physics, University of Patras, Patras, 26504, Greece
[5] FORTH/ICE-HT, Stadiou str Platani, Patras 26504 Greece
[6]Naval Research Laboratory, 4555 Overlook Ave SW, Washington, DC 20375-5320, U.S.A
[7] Department of Physics and Astronomy, University of Sussex, Brighton BN1 9RH, United Kingdom
[8]School of Physics, Department of Solid-State Physics, Aristotle University of Thessaloniki, Thessaloniki,54124, Greece

* Authors to whom any correspondence should be addressed.

Email: sopsilo@iesl.forth.gr, stratak@iesl.forth.gr, and gnk@materials.uoc.gr

Room Temperature Raman characterization of monolayer WS_2

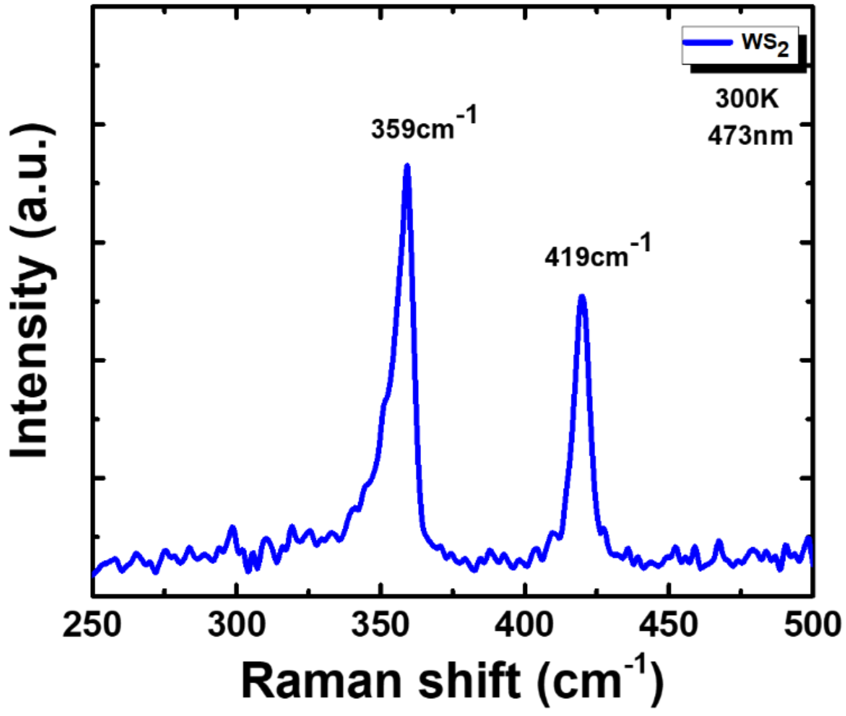

Figure S1: The energy difference of the prominent vibrational modes is 60cm^{-1} which proves the existence of monolayer WS_{2}.

Room temperature photoluminescence characterization for the regions of interest

Figure S2 presents a comparison of photoluminescence (PL) emissions originating from both suspended and strained regions within the same monolayer of WS_2. The impact of Si/SiO_2 on the 2D materials is well known, introducing disorder through factors such as local strain resulting from the amorphous nature of the oxide layer, carrier doping, and impurities. In the context of suspended WS_2, the absence of a substrate beneath contributes to an enhanced PL emission. In contrast, strained areas of WS_2 exhibit a noticeable suppression in PL intensity. The influence of substrate disorder is evident here, as the monolayer comes into contact with the bottom of the cylindrical well. Additionally, strain-induced bandgap narrowing may account for the observed redshift in the PL emission energy in this particular case.

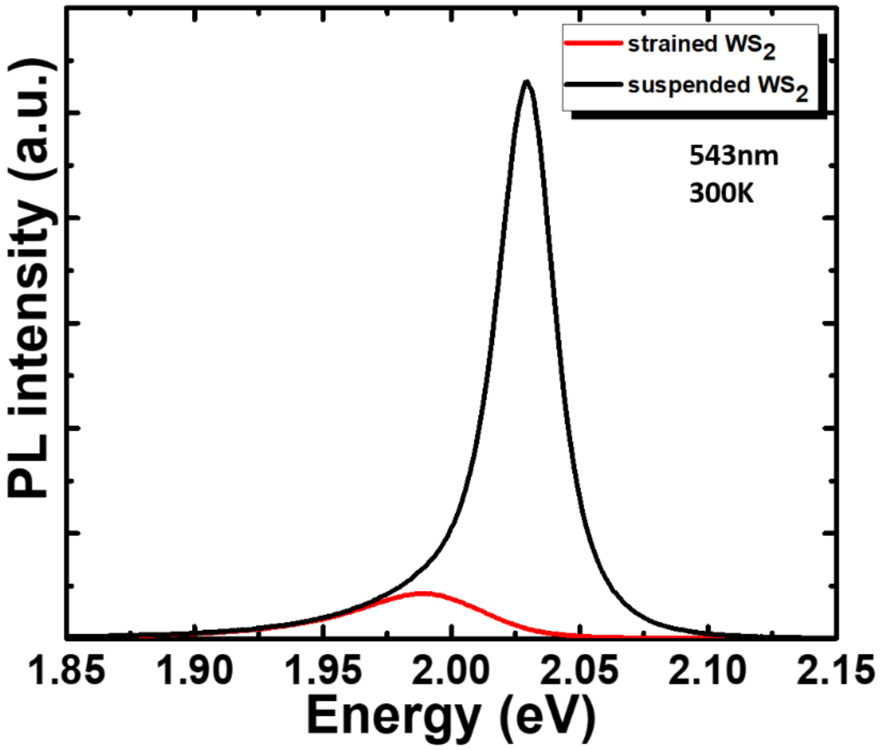

Figure S2: Suspended areas (black line) present a 10-fold enhancement in contrast to strained areas (red line)

Quality of fitting (R^2) mapping of P-SHG measurements

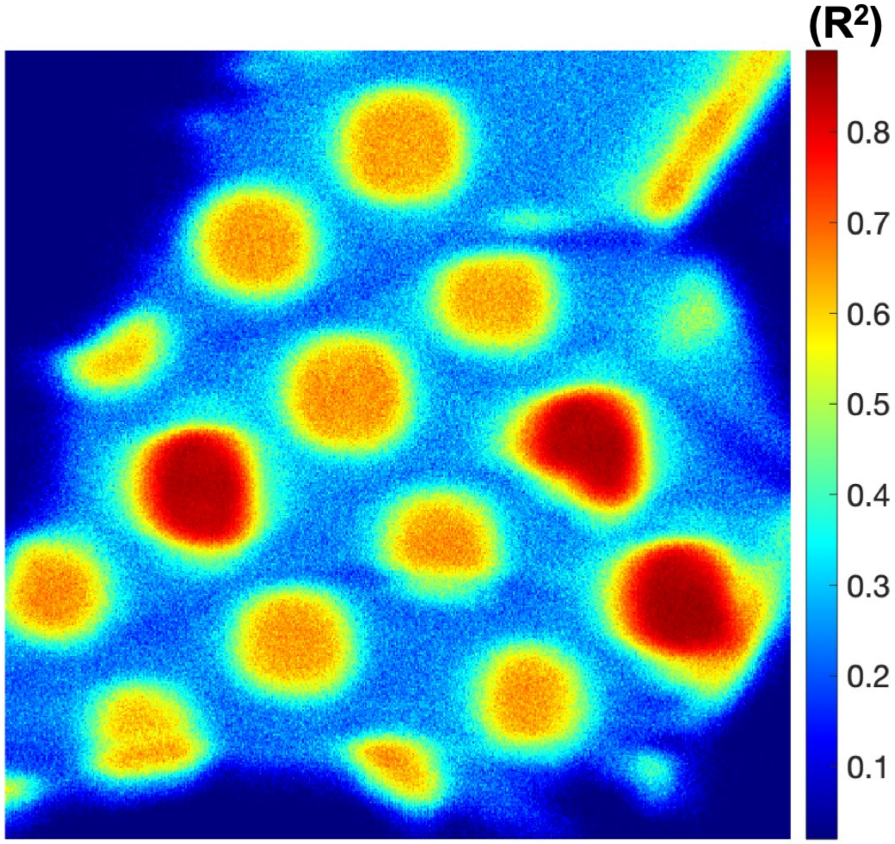

Figure S3: Pixel-wise mapping of R^2 for Fig. 2c.
